# People Living With Dementia and Their Families as Educators for Social Justice

**DOI:** 10.1111/hex.70244

**Published:** 2025-03-27

**Authors:** Clare Mason, Ana Barbosa, Danielle Jones, Michael Andrews, Andrea Capstick

**Affiliations:** ^1^ Centre for Applied Dementia Studies University of Bradford Bradford UK; ^2^ University of Bradford's Centre for Applied Dementia Studies Bradford UK

**Keywords:** dementia, education, film, social inclusion, social justice, training

## Abstract

**Background:**

It is increasingly recognised that dementia education and training should include the direct voices of Experts by Experience (people living with dementia and their families). Good practice in facilitating teaching roles for people living with dementia needs to be identified to maximise inclusion and promote social justice.

**Objective:**

This study aims to discuss the co‐creation of a suite of learning modules on dementia, whose predetermined content was consistent with the three tiers of the UK Dementia Training Standards (DTS), which include filmed interviews with people living with dementia and family members.

**Design:**

Experts by Experience advised on content and took part in filmed interviews contributing to the development of 14 interactive learning modules based on the DTS curriculum. The process was evaluated using (1) participant and facilitator reflection on the film‐making process and (2) independent researcher analysis of the films' content.

**Results:**

Seven people living with dementia and 10 family members took part. Four key points are identified regarding good practice in the co‐creation of film‐based learning materials with people living with dementia and their families. Five key themes are identified from the films' content, highlighting Experts by Experience spontaneous reference to experiences of perceived injustice related to their diagnosis, independently of the intended content of the module.

**Conclusion:**

The active involvement of people living with dementia and their families in practitioner and professional education requires us to pay close attention to what they say. Learning materials should be Expert by Experience‐led rather than curriculum‐led.

**Patient or Public Contribution:**

People living with dementia and their families were involved in the design, conduct and evaluation of the study and in the preparation of the manuscript.

## 1 Introduction

The involvement of patients, their families and the public – also known as Patient and Public Involvement and Engagement (PPIE) – in UK health and social care has grown significantly in recent decades. These developments coincide with an increased recognition of the role of Experts by Experience (EbyE) in improving practice and patient outcomes [1, 2] and a shift towards a more human rights‐based approach (HRBA) to service provision.

Over the years, a range of policies and legislation have placed EbyE at the centre of health and social care provision. Of relevance, the White Paper *Equity and Excellence: Liberating the NHS* [3], highlighted the importance of patients and the public by embedding the principle ‘Nothing about us without us’, which supported the view that people should participate in decisions and policies that affect their care. The subsequent consultation work *Liberating the NHS: An Information Revolution* [4] developed this further, promoting a national drive to give people control and choice. As a result, it has become increasingly common for EbyE insights to inform the planning, commissioning, delivery and evaluation of the care services they receive.

There has been a parallel move to increased involvement of EbyE in health and social care education and training, where it is now widely recognised that the expertise and experience of EbyE should be at the core of any education opportunities for health and social care professionals [5]. The role of EbyE in raising awareness of issues that are important to them and contributing their lived experience is known to improve the quality of education and to support health and social care professionals to change practice [5, 6].

The involvement of people living with dementia as EbyE in education and training seems to be lagging behind by comparison with other aspects of PPIE such as co‐creative research and critical analysis of current standards of service delivery [7, 8]. It is noticeable in the literature that most reports of involvement activity focus on general mental health training [9, 10]. A notable exception is the involvement of four people living with dementia in the delivery of a Foundation Degree programme at a UK University [2]. Additionally, 22 people living with dementia and their families participated in the design, teaching and assessment of a postgraduate module delivered by the co‐authors of this paper (C.M. and D.J.). Elsewhere, however, people living with dementia have historically been excluded from decisions about their lives because they are seen as unable to communicate effectively or as unreliable in sharing their experiences [11].

In parallel contexts, this tendency to disbelieve or discredit personal testimony has been described as ‘epistemic injustice’ [12]. The dementia advocacy movement has been central in challenging these assumptions and promoting active rather than passive involvement of people living with dementia [13, 14]. The move towards a more HRBA to dementia [15] now demands that we also rethink policy and practice on how those living with dementia contribute to all aspects of education and training.

Absence of EbyE involvement or tokenism in dementia education and training was noted as a problem more than 15 years ago [16], yet as recently as 2017 a literature review on the most important components of dementia education [17] fails to include the involvement of people living with dementia. The onus should, however, lie with researchers, educators and service providers to adapt their methods to make the active inclusion of people with differing cognitive abilities possible, rather than including only an unrepresentative minority [7]. In research contexts, it has been noted, for example, that standard ‘sit‐down’ interviews are often an uncomfortable means for creating data with people living with dementia [18]. This point has been reiterated by people living with dementia themselves [8] and is equally likely to be true in educational contexts. PPIE is complex and multi‐faceted and cannot be assumed to be an automatic corrective to exclusionary practices [19].

We suggest in this article that drawing on some of the principles of education for social justice may help to ensure that the involvement of EbyE is authentic and meaningful rather than ad hoc and tokenistic.

### 1.1 Education for Social Justice

The term education for social justice refers to education that attempts to incorporate teaching about social inequalities rather than ignoring them. We believe it is important to apply theory related to education for social justice in this context because PPIE in education for people living with dementia and their families is not simply a matter of taking part; it should also be a question of influencing what is taught. This introduces a different perspective, highlighting that many behaviours of people living with dementia are not solely due to cognitive impairment but also responses to psychological and social factors, such as others' reactions and the suitability of services. Many of these responses can be seen as forms of cultural resistance [20].

The two main dimensions of education for social justice we are concerned with here are

a. Access: Social justice in *access* to education is often discussed in relation to students, particularly in how disadvantaged social groups have less access to non‐mandatory education [21]. However, this concept can also apply to those who can take on teaching roles. Historically, people living with dementia have had limited opportunities to occupy such roles. This article aims to explore the importance of including people with dementia as teachers, and how their involvement can be supported and facilitated.
b. Curriculum: Social justice in the *curriculum* occurs when students are encouraged to think differently about issues such as discrimination, social inequality, stigma and social exclusion, and when both teachers and learners reflect on the impact of social injustice on their own lives [22].

### 1.2 The Dementia e‐Learning Programme

In recent years, there has been increasing demand for health and social care professionals with specialist knowledge and skills in dementia care. In 2015, the Department of Health commissioned the Dementia Core Skills Education and Training Framework, developed with Skills for Health, Health Education England (HEE) and Skills for Care. Updated in 2018 as the Dementia Training Standards (DTS) framework, it defines three tiers of training: awareness (Tier 1), practical skills (Tier 2) and leadership (Tier 3), ensuring consistent dementia training across health and social care. It includes expected learning outcomes for training delivery, and it aims to help ensure the quality and consistency of dementia training.

HEE's e‐Learning for Healthcare first commissioned the co‐authors' university in 2015 to develop online training aligned with the framework. In 2019, the university was re‐commissioned to review and update the sessions (Box 1).

Box 1. **Dementia e‐learning modules**

- Dementia awareness (Tier 1)
- Person‐centred dementia care (Tier 1)
- Dementia identification, assessment and diagnosis (Tier 2)
- Dementia risk, reduction and prevention (Tier 2)
- Communication, interaction and behaviour in dementia care (Tier 2)
- Health and well‐being in dementia care (Tier 2)
- Pharmacological interventions in dementia care (Tier 2)
- Living well with dementia and promoting independence (Tier 2)
- Families and carers as partners in dementia care (Tier 2)
- Equality, diversity and inclusion in dementia care (Tier 2)
- Law, ethics and safeguarding in dementia care (Tier 2)
- End‐of‐life dementia care (Tier 2)
- Research and evidence‐based practice in dementia (Tier 2)
- Leadership in transforming dementia care (Tier 3)

Online learning is often the only option available for health and social care professionals requiring specialised dementia education. It provides flexible, personalised learning, overcoming barriers of distance and time while catering to diverse practice settings [23]. Over the last decade, its popularity has grown, becoming essential during COVID‐19. Evidence supports its effectiveness; a recent scoping review found online education improved access and equity for social workers, with performance and satisfaction comparable to face‐to‐face learning [24]. The impact of this Dementia e‐learning programme on health and social care professionals' knowledge, attitudes and confidence has been published elsewhere [25].

## 2 Aims

To examine the involvement of people living with dementia and their families in a specific Dementia e‐learning programme with reference to principles of education for social justice including issues of (a) access to, and facilitation of, educational roles, (b) influence over the curriculum.

## 3 Methods

The Dementia e‐learning programme was developed by a multidisciplinary team with backgrounds in psychology, social work, gerontology and speech and language therapy. Some team members also had experience as carers and had previously worked in dementia support services. They applied research evidence to design content aimed to be transferable and applicable across different types of service provision. EbyE were involved in the design of the suite of 14 e‐learning modules. The topics for the modules were set by the framework (Box 1). EbyE were assigned to modules aligned with their expertise. Module authors drafted initial content, which was then shared with EbyE for feedback. We worked with some individuals in person to review the modules, while others received printed versions or online links for their input. Changes were made based on their feedback. EbyE also took part in filmed interviews which were embedded within the modules. Film was used intentionally as a method for promoting the social inclusion of people living with dementia, ensuring that they were seen as well as heard. The film‐making process is thus detailed in this manuscript.

### 3.1 Recruitment

EbyE were recruited to the project from the Centre for Applied Dementia Studies EbyE group and wider networks. The EbyE group is currently made up of 150+ people living with dementia and family members, including unpaid current and former carers. At the time, it had 58 members. A flyer was created advertising the opportunity to be involved in reviewing existing materials and creating new ones for the e‐Dementia e‐learning programme. The information was distributed by post and emailed to those on the group database and advertised on social media platforms such as X and Facebook. The flyer was also electronically distributed amongst professional networks and hard copies were sent to the local memory clinics and post‐diagnostic support service providers. Those who expressed an interest were provided with further information and any queries were answered by email, telephone, or in person, depending on individual needs and preferences.

An information sheet that had been reviewed by an EbyE covering the project aims, payment for involvement and travel expenses, and the consent process, were distributed both by email and hard copy.

### 3.2 EbyE

Out of 32 expressions of interest, 17 EbyE were recruited: seven people with dementia (five males, two females) and 10 family members (three males, seven females). Diagnoses included young‐onset dementia (2), Alzheimer's disease (2), vascular dementia (1), mixed dementia (1) and posterior cortical atrophy (1). Ages ranged from 59 to 75 for people with dementia and 26–89 for family members. Fifteen individuals were White British, two White Irish. Two couples participated; the others joined individually. Twelve individuals were recruited from the EbyE group, and five from a local dementia support group.

### 3.3 Film‐Making Process

The PPIE lead and academic project lead liaised with the University's Film School, to discuss what was needed for each module and how this might be achieved. The PPIE lead also regularly liaised with the cinematographer who would be doing the filming.

Before the filming began, the PPIE lead provided the film‐making team with an overview of each EbyE, drawing on their in‐depth knowledge of the individuals. This ensured that any additional needs, such as mobility or communication support, could be appropriately addressed. One couple, for example, was unable to leave their home due to other caring responsibilities and was visited and filmed at home. Careful consideration was also given to verbal prompts that might be used to elicit this experience, and the level of support each person might need. Discussions were also held on the location of interviews, about the set‐up of the cameras and sound equipment, and how this might work alongside the needs of the people being interviewed.

Module authors and EbyE co‐designed the module content and decided on priorities for practitioner education. Together they identified where video content might enhance the written content. These discussions were used to create a set of interview prompts to be used during filming (see Box 2 for an example).

Box 2. **Example of Interview Questions for ‘Dementia Identification, Assessment and Diagnosis’**

- Can you tell me about your diagnosis?
- Tell me what it felt like getting a diagnosis?
- What were your first thoughts then? After you were given that diagnosis?
- Thinking back then to how you were given the news that you'd got your diagnosis, what could have been done differently?
- I wonder though, how would you have felt your family knowing before you did?
- Had you been told anything about it being a life‐threatening illness?

Filming took place at the University in a quiet room. Refreshments were provided and toilet facilities were also close by. Some EbyE could attend on some of the filming dates but not others, meaning a timetable had to be drawn up, allowing a gap between each interview for participant crossover. We also took into consideration the time of day when EbyE were more alert, knowing that if people were rushed or felt anxious, this would impact the quality of the interviews. We were also flexible if arrangements needed to be changed at short notice (e.g. due to hospital appointments). On the day of filming, an explanation was given of what would happen, including that the interview could be stopped at any time. Practical support was offered, which included the management of the cables associated with the camera, position and adjustments to the lighting. EbyE were filmed talking about their experience of the aspect of dementia covered by the module in question. At the end of the interview, those who needed help were accompanied off campus.

The length of film clips ranged from a few seconds to several minutes. When the films had been edited, each person was shown their film, either by an online link or DVD. Any changes, queries, comments, or concerns were noted and acted upon where possible; for example, captions were adjusted to reflect the ways EbyE wished to describe themselves. A screening was arranged with a meal at the local City Hall, at which each participant received a copy of their film, a thank you card and a voucher. Payment was aligned with the National Institute for Health and Care Research (UK government agency) guidance.

### 3.4 Data Analysis

Data analysis took place in two phases related to:

1. The film‐making process: evaluation to ascertain strengths and areas for improvement for future involvement of people living with dementia and their families in PPIE related to education and training;
2. The content of the films: examination of the films' content to identify any departures from the originally intended curriculum.

For (1), an initial developmental evaluation of the film‐making process was carried out by making aide‐memoire style notes during filming as a record of any problems that arose. The notes taken were used to identify salient topics and these were cross‐checked by the team. Notes were supplemented by the interviewer's more detailed reflections on the process. Later, EbyE views and those of the film‐maker were also collected and incorporated into the evaluation. This material was used iteratively to improve the process as the film‐making proceeded and has been used in all filming carried out since. For example, the interviewer noted, in respect of facilitating communication:

Questions were rephrased where appropriate. I also learned to use more gestures, such as nodding, positive facial expression (smiling etc) to emphasise when a person was ‘doing well’ and to encourage them to continue…

For (2), content analysis of the film clips embedded within the 14 modules was later carried out by a researcher (C.M.) to assess the extent to which EbyE's contributions corresponded with, or deviated from, the intended content of the module. Instances of EbyE subverting the intended module content by introducing their perspectives on, for example, the cause of a problem or non‐biomedical factors exacerbating the experience of dementia were extracted and divided into key themes, paying particular attention to experiences of social injustice and human rights' issues.

## 4 Ethical Procedures

Ethical approval was not obtained for this project, as it involved PPIE activities where individuals acted as advisors rather than research subjects. EbyE received clear information about the project, including its purpose, potential risks, benefits and their right to withdraw without consequence. Verbal consent was obtained before participation. Pseudonyms were used, and data was securely stored on password‐protected systems with restricted access. Quotes in this study were taken from publicly available modules and used with permission.

## 5 Findings

### 5.1 Key Findings From the Film‐Making Process

The data has been organised into four key topics based on the interviewer's notes, as well as the views of EbyE and the film‐maker. These topics highlight the challenges encountered in co‐creating film‐based learning materials with people living with dementia and their families, and form the basis for practice recommendations.

### 5.2 Environmental Preparedness

Despite prior assurances about soundproofing, filming was frequently interrupted by noise from students, external repair work and planes overhead. These disruptions not only affected the quality of the film but also impacted EbyE concentration and anxiety levels. However, these interruptions provided opportunities to engage EbyE in discussions about their preferences for a conducive filming environment, enhancing their agency in the process.

### 5.3 Interviewing Timing and Design

Initially, insufficient time was allocated to each interview, for EbyE with dementia to process information and formulate responses. Questions often needed rephrasing or repetition due to short‐term memory issues, which extended interview times and impacted overall schedules. This experience highlighted the importance of allowing ample time for EbyE to engage fully, leading to richer, more thoughtful responses. In addition, the variability in EbyE's responses highlighted the need for open‐ended questions to elicit more detailed answers. Some EbyE provided quick, short answers, while others required more prompting to share their experiences. This highlights the need for questions to also be adaptable to different communication styles and capable of drawing out comprehensive narratives from EbyE.

### 5.4 Managing Interpersonal and Logistical Challenges

Some couples were interviewed together, revealing differing perspectives on the questions asked. This occasionally led to distress, particularly when one partner disclosed upsetting opinions in the presence of the other. However, these interactions also highlighted the value of capturing diverse viewpoints, which enriched the learning materials by presenting a more nuanced understanding of living with dementia. Some last‐minute logistical adjustments on the day of filming, such as a partner remaining present during an interview, also caused additional interruptions. The interview became quite stilted, and the person with dementia lost their train of thought as a result. These experiences highlight the need for clear communication, flexibility in planning, and the ability to mediate sensitively. Facilitators should be prepared to address unexpected changes and interpersonal dynamics, ensuring EbyE comfort and consent are prioritised.

### 5.5 Emotional Support and Follow‐Up

EbyE sometimes shared deeply emotional experiences, which could be distressing for both the interviewee and the interviewer. Several EbyE became tearful during interviews, necessitating unplanned follow‐up to provide emotional support and guidance. These practices reinforced the commitment to participant well‐being and the importance of having support mechanisms in place, such as follow‐up sessions and access to resources like Carers' Resource and Alzheimer's Society Talking Point, and Admiral Nurses. In addition, an emotional response from an untrained crew member highlighted the need of providing comprehensive training in dementia awareness and emotional support for all staff.

### 5.6 Social Injustice – Findings From Content Analysis of the Films

The previous section identifies several improvements that could be made to ensure the process used to involve EbyE in making the films is socially inclusive. We also need to reflect, however, on the social justice of hearing what people living with dementia and their families want to say and acting on it, including any concerns they disclose about current injustices. The questions that were asked during the interviews focused on the topic of the module in question. There was considerable emphasis on problems people experienced because of dementia, and fewer prompts related to non‐biomedical issues such as stigma, or the impact of other people's lack of awareness. Nevertheless, it was noticeable that EbyE frequently subverted the intended subject area of the module in question and discussed instead their own views on what helped or hindered them in day‐to‐day life. These experiences were often ones of social injustice rather than symptomatology, and fell into five main areas, which are briefly discussed below with examples:

- Stigma and social exclusion
- Lack of awareness or poor communication by others
- Risk averseness on the part of professionals
- The financial impact of dementia
- Insults to dignity and respect

Of these, only communication is overtly included in the DTS Framework subject areas and here the focus is on problems with communication resulting from dementia, rather than the poor communication on the part of others that EbyE were keen to discuss.

*Stigma and social exclusion*: Several EbyE spoke of the excess disability that resulted from being socially isolated, particularly when they were not able to go out. For example, Tony, in the module on Dementia Awareness, suggests that memory problems have as much to do with social isolation as with dementia:

I need to get out. I've got to mix with people. I find if…for some reason, say, I can't get out of the house, I think it makes me worse. I'll, I'll start forgetting things or forget to do things. [Tony, person with dementia]

Sandra noted that stigma attached to a dementia diagnosis, arose in her case from dementia being viewed – inaccurately – as a terminal disease.

My manager even said, ‘How long have you got?’ So, that made me think, ‘Oh well maybe I haven't got long’, you know. It brought me down mood‐wise greatly. [Sandra, person with dementia]

*Lack of awareness or poor communication by others*: EbyE mentioned numerous examples of unclear or inappropriate communication by others. In some instances, this amounted to bullying by those in positions of authority. In relation to a consultation with a psychiatrist described in the module Dementia Identification, Assessment and Diagnosis, one participant said, for example:

He absolutely belted at me with questions, and when you got a problem like dementia, …somebody coming at you with an ack‐ack gun type of questioning, you can't cope with it…I said, “I want to try and explain”… and this is the truth of how he dealt with me, he said, “All I want from you is yes or no, I don't want you to sit there and waffle at me.” [Duncan, person with dementia]

In other cases, basic information had not been conveyed to relatives. This was mentioned by Valerie in the module on End‐of‐life Care:

I worked for an advocacy place and the man that was the head of it was a nurse… I said, ‘Mum keeps blinking and going cross‐eyed’ and he said, ‘She's having mini strokes’. I said, ‘Is she? Nobody's told me. Is that part of Alzheimer's?’ He said, ‘No, not really… it's vascular’. And I was never made aware that it was vascular dementia until the death certificate came. [Valerie, carer]

*Risk averseness on the part of professionals*: Some EbyE referred to being inappropriately supervised by risk‐averse professionals so that their freedom of movement was, or was in danger of being, curtailed. This extract is taken from the module on Dementia Identification, Assessment and Diagnosis:

I was a lorry driver, and I'd planned to carry on until basically I dropped or until they tell me no you can't pass the medical, I was expecting to get work until I'm in my 70 s. And then they took my licence off me… car licence, bike licence, lorry licence. And when I asked, “Can I not even ride a bike?” “No we wouldn't even let you on a push bike…” and I thought “That's it… I'm finished”. [Martin, person with dementia]

In other cases, it was a lack of flexibility on the part of professionals that was perceived to be the problem. In this extract, whilst staying close to the intended subject of the module, Pharmacological Interventions, Sandra notes an imbalance of power between professional experts and those whose expertise has come from experience:

It was only when I started on Twitter and I put a message out to Twitter, “Can anybody help me cos I'm having these terrible hallucinations on Aricept” The one I remembered most was “Just take it in the morning, don't take it at night.” The pharmacists want you to take it at night but the Twitter said “Try taking it in a morning”, so I now I have all my tablets in a morning, and I don't have hallucinations. It was as quick as that, it was an overnight change. Um, but again no one tells you that because the rules say you must take it in the evening. No flexibility. [Sandra, person with dementia]

*The financial impact of dementia*: Closely tied in with the loss of paid employment were experiences EbyE related to the financial impact of a diagnosis of dementia, a topic neglected in the literature. In the module on Dementia Awareness, Sandra discusses the financial aspects of living with young‐onset dementia:

I was living alone, so the mortgage was my mortgage and [it was] me who paid the bills, and I realised there's no one to go to for financial advice for people with young‐onset…I couldn't reduce my hours because I wouldn't be able to pay my mortgage …so I had to retire to get my pension, to be able to pay off my mortgage. You don't think of anything like that when you're given a diagnosis. [Sandra, person with dementia]

In the module on Law, Ethics and Safeguarding, Martin talks about the impact a social worker's assumptions had on aspects of his autonomy such as living alone and managing his own finances:

He had this impression if you've got dementia you can't live on your own, you should be in care. And then he actually brought somebody else in. This bloke phoned me and he says “How are you coping with your bills?” I said “I've got everything sorted out.” “Well how do I know you're paying them? From now on I want to see all your mail” he says. “Next week when I come I want you to give me all your mail”. I said, “Right no problem.” And when he came I threw down all the junk mail… pizzas and curry houses. He says “What's that?” and I said, “All the mail I had.” [Martin, person living with dementia]

*Insults to dignity and respect*: Deficits in basic physical care for people with more severe dementia often meant that their personal dignity was in danger of being compromised. As in the extract below, seeing that justice is done often demands extraordinary determination:

I remember saying 1 day to the care home, ‘I'm not taking my mum‐in‐law out without her teeth and I can't find them’. She would never have gone out without them… and I was determined that we weren't paying for them. I said, ‘You'll have to claim them on the insurance; you must have insurance’ and they said, ‘Yes, but they're not lost’. So I said, ‘Well, unless you can find them they are lost’. So they did claim them, and she got a new set of teeth. [Val, carer]

Notably, this excerpt is from the end‐of‐life care module, which diverges from its intended focus by discussing Valerie's mother‐in‐law's enjoyment of outings. In the following section, we discuss how both the filmmaking process and the content of these films influence EbyE's involvement in promoting social justice within dementia education.

## 6 Discussion

This article adds to the literature on EbyE involvement in dementia education by suggesting two key components. The first is accessibility which involves enhancing the conditions under which people who are living with dementia and family members contribute to the design, delivery and evaluation of educational materials aimed at practitioners and professionals. The second is a question of having genuine influence over what is taught and how it is taught.

The findings reported highlight what works well when involving people living with dementia in practitioner education and what to avoid. There are many practical, ethical and methodological issues to be addressed at the point of initial input to codesign and co‐creation. Having a setting away from any potential disruptions, such as sources of noise, is essential, as is individually adapting elicitation techniques. Standard interview techniques may not be the best way of eliciting information; but there are many adaptations that can be made such as recording and transcribing informal discussion groups [7, 18]. Being alert to interpersonal dynamics and understanding the potential for emotional distress relating to sensitive subjects is also crucial. When EbyE are asked to discuss personal experiences, this can often be upsetting, and we also need to consider who is a party to any disclosures that are made. Some people may prefer group discussions whilst others may need one‐to‐one support. Differences of opinion are to be welcomed, and they help practitioners to understand heterogeneity among people living with dementia. They can also help to demonstrate that family caregivers' views do not necessarily correspond with those of the person with dementia they are supporting. However, involvement should not leave anyone feeling hurt and extra support staff and basic dementia awareness training are essential. Allowing plenty of time (double the duration you originally thought of is a good rule of thumb) and avoiding changes at the last minute are also recommended.

Much of the focus so far has been on practical aspects of PPIE in dementia education, rather than the larger issue of how much influence people with dementia have on curricula [8]. Education that merely seeks the support of EbyE for an existing curriculum is tokenistic [16]. Genuine input from people with dementia must be listened to and acted upon. To promote education for social justice in the dementia field we therefore need to ensure that EbyE not only have *access* to teaching roles [21] but also that they influence what is taught [22]. Achieving epistemic justice [12], as opposed to injustice, for people living with dementia and their families, requires more than participation; it needs attentiveness to what they say, and encourage practitioners to act upon it. In this way, practitioners may also become more generally aware of social injustice, including how this impacts their own lives in the workplace and in broader society [22]. ‘Recovery colleges’ [26] are a pioneering example of coproduction in education. This approach brings together EbyE and healthcare professionals to cocreate and deliver courses. This ensures education is inclusive, equitable and truly reflects the voices of those it serves, setting a standard for promoting social justice in dementia education. EbyE can significantly shape the educational agenda and open up new areas that need to be addressed. Even when EbyE were not directly asked about instances of social injustice, they were keen to identify instances of injustice where an HRBA [15] was not in evidence. Education for social justice involves acknowledging that people living with dementia and their families are knowers, educators and experts on their own experiences, that such experiences are diverse, and that they do not result solely from dementia. By embedding the lived experiences of EbyE through video testimonials, we have offered real‐world insights encouraging learner reflection and discussion on social injustice. They have directly shaped the curriculum by prompting the inclusion of supplementary resources, supporting learners in exploring additional readings that deepen their understanding of the issues raised.

*Limitations of the work*: Because this study was externally commissioned to an existing brief, and carried out within a set timeframe, there has been considerable learning in the process. With hindsight, as discussed above, the traditional interview format used to create the learning materials was not ideal for many of the people who took part. There could also have been greater diversity among the EbyE in terms of age, living arrangements and ethnicity. Although individuals from different backgrounds were invited, their lack of participation suggests a need to actively seek a broader range of EbyE by reaching out through diverse channels and community organisations specifically serving diverse populations. The research centre where the study was based has since gone on to develop learning materials which are co‐created and delivered by people living with dementia. While the design of this study was not created with PPIE input, an EbyE is a co‐author and helped us shape and revised this manuscript. For authentic co‐creation, it would be valuable to include EbyE in the study design process from the outset.

## 7 Conclusion

Merely supporting the involvement of people living with dementia in practitioner education is not enough. EbyE need to be recognised as having a direct role in teaching, including setting curricula. Adjustments may often need to be made to ensure that EbyE have a positive experience of taking on an educational role. By role modelling reasonable adjustment, educators may encourage other organisations and employers – who often too quickly assume that a dementia diagnosis is the end of an employee's working life ‐ to do the same. To learn from people living with dementia, practitioners need to be encouraged to engage with and analyse what EbyE educators have to say, rather than merely meeting learning outcomes identified in advance as part of an official curriculum. Human rights‐based dementia education should therefore shift the balance of power towards those whose rights are most directly affected. We can start by ensuring that EbyE are able to have a genuine influence over what is taught.

## Author Contributions

**Clare Mason:** conceptualisation, investigation, writing – original draft, writing – review and editing, project administration, methodology. **Ana Barbosa:** writing – original draft, writing – review and editing, formal analysis, supervision. **Danielle Jones:** writing – review and editing. **Michael Andrews:** writing – review and editing. **Andrea Capstick:** conceptualisation, writing – original draft, writing – review and editing, formal analysis.

## Conflicts of Interest

The authors declare no conflicts of interest.

## Data Availability

The data that support the findings of this study are available from the corresponding author upon reasonable request.

## References

[1] V. Cluley , A. Ziemann , C. Feeley , E. K. Olander , S. Shamah , and C. Stavropoulou , “Mapping the Role of Patient and Public Involvement During the Different Stages of Healthcare Innovation: A Scoping Review,” Health Expectations 25, no. 3 (2022): 840–855. 35174585 10.1111/hex.13437 PMC9122470

[2] C. W. Russell , “People With Dementia, Contributing to Learning and Teaching in Higher Education: Innovative Practice,” Dementia 19, no. 2 (2020): 447–452. 28024419 10.1177/1471301216685460

[3] Department of Health , Equity and Excellence: Liberating the NHS, Vol. 7881 (The Stationery Office, 2010).

[4] Department of Health , Liberating the NHS: An Information Revolution (Department of Health, 2010).

[5] B. Kuti and T. Houghton , “Service User Involvement In Teaching and Learning: Student Nurse Perspectives,” Journal of Research in Nursing 24, no. 3–4 (2019): 183–194. 34394524 10.1177/1744987119837594 PMC7932287

[6] K. S. Pallesen , L. Rogers , S. Anjara , A. De Brún , and E. McAuliffe , “A Qualitative Evaluation of Participants' Experiences of Using Co‐Design to Develop a Collective Leadership Educational Intervention for Health‐Care Teams,” Health Expectations 23, no. 2 (2020): 358–367. 31999883 10.1111/hex.13002 PMC7104638

[7] A. Capstick , A. Dennison , J. Oyebode , et al., “Drawn From Life: Cocreating Narrative and Graphic Vignettes of Lived Experience With People Affected by Dementia,” Health Expectations 24, no. 5 (2021): 1890–1900. 34378295 10.1111/hex.13332 PMC8483207

[8] J. McKillop and F. Kelly , “A Social Citizenship Lens to Describe One Person's Experience of Living With Dementia in Scotland,” in Everyday Citizenship and People Living With Dementia (Dunedin Academic Press, 2019).

[9] B. Happell , T. Warner , S. Waks , et al., “Something Special, Something Unique: Perspectives of Experts by Experience in Mental Health Nursing Education on Their Contribution,” Journal of Psychiatric and Mental Health Nursing 29, no. 2 (2022): 346–358. 34032356 10.1111/jpm.12773

[10] A. Horgan , F. Manning , J. Bocking , et al., “To Be Treated as a Human’: Using Co‐Production to Explore Experts by Experience Involvement in Mental Health Nursing Education – The COMMUNE Project,” International Journal of Mental Health Nursing 27, no. 4 (2018): 1282–1291. 29377483 10.1111/inm.12435

[11] J. Bethell , E. Commisso , H. M. Rostad , et al., “Patient Engagement in Research Related to Dementia: A Scoping Review,” Dementia 17, no. 8 (2018): 944–975. 30373460 10.1177/1471301218789292

[12] M. Fricker , “Evolving Concepts of Epistemic Injustice.” The Routledge Handbook of Epistemic Injustice (Routledge, 2017), 53–60.

[13] T. Davies , A. Houston , H. Gordon , et al., “Dementia Enquirers: Pioneering Approaches to Dementia Research in UK,” Disability & Society 37, no. 1 (2022): 129–147.

[14] K. Seetharaman and H. Chaudhury , “‘I Am Making a Difference’: Understanding Advocacy as a Citizenship Practice Among Persons Living With Dementia,” Journal of Aging Studies 52 (2020): 100831. 32178801 10.1016/j.jaging.2020.100831

[15] C. Bryden , Nothing About Us, Without Us!: 20 Years of Dementia Advocacy (Jessica Kingsley Publishers, 2015).

[16] S. Cahill , “New Analytical Tools and Frameworks to Understand Dementia: What Can a Human Rights Lens Offer?,” Ageing and Society 42, no. 7 (2022): 1489–1498.

[17] K. Hope , D. Pulsford , R. Thompson , A. Capstick , and T. Heyward , “Hearing the Voice of People With Dementia in Professional Education,” Nurse Education Today 27, no. 8 (2007): 821–824. 17698260 10.1016/j.nedt.2007.06.005

[18] C. A. Surr , C. Gates , D. Irving , et al., “Effective Dementia Education and Training for the Health and Social Care Workforce: A Systematic Review of the Literature,” Review of Educational Research 87, no. 5 (2017): 966–1002. 28989194 10.3102/0034654317723305 PMC5613811

[19] R. Bartlett and D. O'Connor , Broadening the Dementia Debate: Towards Social Citizenship (Policy Press, 2010).

[20] F. Poland , G. Charlesworth , P. Leung , and L. Birt , “Embedding Patient and Public Involvement: Managing Tacit and Explicit Expectations,” Health Expectations 22, no. 6 (2019): 1231–1239. 31538704 10.1111/hex.12952 PMC6882252

[21] F. Polat , “Inclusion in Education: A Step Towards Social Justice,” International Journal of Educational Development 31, no. 1 (2011): 50–58.

[22] E. Smith , Key Issues in Education and Social Justice (Sage, 2018).

[23] Skills for Health , Dementia Training Standards Framework. Skills for Health, Health Education England and Skills for Care 2018.

[24] D. Button , A. Harrington , and I. Belan , “E‐Learning & Information Communication Technology (ICT) in Nursing Education: A Review of the Literature,” Nurse Education Today 34, no. 10 (2014): 1311–1323. 23786869 10.1016/j.nedt.2013.05.002

[25] S. Parveen , S. J. Smith , C. Sass , et al., “Impact of Dementia Education and Training on Health and Social Care Staff Knowledge, Attitudes and Confidence: A Cross‐Sectional Study,” BMJ Open 11, no. 1 (2021): e039939. 10.1136/bmjopen-2020-039939 PMC7817792 33468498

[26] E. Wolverson , L. Hague , J. West , et al., “Building an Initial Understanding of UK Recovery College Dementia Courses: A National Survey of Recovery College and Memory Services Staff,” Working With Older People 28, no. 2 (2023): 108–119.

